# Home-based telemonitoring versus hospital admission in high risk pregnancies: a qualitative study on women’s experiences

**DOI:** 10.1186/s12884-020-2779-4

**Published:** 2020-02-04

**Authors:** J. F. M. van den Heuvel, C. J. Teunis, A. Franx, N. M. T. H. Crombag, M. N. Bekker

**Affiliations:** 1 Department of Obstetrics, University Medical Center Utrecht, Utrecht University, Ref: KE 04.123.1 Heidelberglaan 100, 3584 CX Utrecht, The Netherlands; 2 Department of Development and Regeneration, Leuven, KU Belgium

**Keywords:** High risk pregnancy, Telemedicine, Pregnancy complications, Fetal monitoring, Patient centered care, Perinatal care

## Abstract

**Background:**

Hospital admission during pregnancy complications is considered to be an event of significant impact. Besides conventional in-clinic maternal and fetal monitoring, recent technologies enable home-based telemonitoring with self-measurements in high risk pregnancy. This study is part of a feasibility pilot to explore the usability and acceptability of telemonitoring and aims to gain insight in the experiences and preferences of high risk pregnant women concerning the novel strategy of telemonitoring, opposed to women who were hospitalized in pregnancy.

**Methods:**

Using secured Facebook Groups, we conducted four online focus groups: two focus groups with women who were admitted during pregnancy (*n* = 11) and two with women who received home telemonitoring in the pilot phase (*n* = 11). The qualitative data were analyzed thematically.

**Results:**

Four major themes emerged from both participant groups: [1] care experience, [2] emotions regarding pregnancy, [3] privacy and [4] impact on daily life. Different views were reported on all four themes, resulting in a direct comparison of experiences during hospitalization and telemonitoring. Most admitted patients reported a growing sense of boredom and anxiety during their clinical admission. Lack of privacy on ward was a great concern, as it affected their contact with hospital staff and family. This issue was not reported amongst telemonitored women. These participants still felt like a patient at times but responded that the comfort of their own home and bed was pleasant. Only a minority of telemonitored participants reported being anxious at times at home, while not having a physician or nurse nearby. Being at home resulted in less travel time for partners or family for hospital visits, which had its positive effects on family life.

**Conclusions:**

Telemonitoring of a high-risk pregnancy provides an innovative manner to monitor fetal and maternal condition from home. Compared to the experiences of hospital admission in high risk pregnancy, it allows women to be in a comforting and private environment during an anxious time in their lives. As future studies should further investigate the safety and cost effectiveness of this novel strategy, women’s views on the preference of telemonitoring need to be taken into consideration.

## Key message

In high risk pregnancy, home-based telemonitoring of maternal and fetal parameters seems to be an acceptable and comfortable manner of antenatal care, based on experiences of two groups of women with either hospital admission or telemonitoring.

## Background

Worldwide, the number of women at increased risk for complications in pregnancy continues to grow due to unhealthy lifestyle, obesity, advanced maternal age at conception and concurrent comorbidities [1–3]. High-risk pregnancy is defined as any pregnancy in which there is a factor—maternal or fetal— that potentially acts adversely to affect the outcome of pregnancy, for example preterm rupture of membranes (PROM), fetal growth restriction (FGR) and preeclampsia (PE) [4]. International guidelines recommend increased monitoring and observation of maternal and fetal parameters, which essentially leads to hospital admittance [5–7].

Hospital admission during pregnancy is considered to be an event of significant impact, because of combined stressors of both pregnancy and hospitalization [8]. In previous quantitative studies on hospitalization during high risk pregnancy, women report lower self-esteem, greater anxiety and depression and less optimal family functioning [9]. Experienced fear, anxiety for the unknown and perceived immobility and inactivity are amongst stressors and emotions during hospitalization [10–12].

Besides conventional care during clinical admission, recent technological advances resulted in e-Health, defined as health services and information delivered or enhanced through the Internet and related technologies [13]. Potential positive effects of the use of *e-Health* include increased patient engagement and satisfaction, better access to health care and the possibility to reduce clinic costs with equal or better health outcomes [14, 15]. *e-Health* has already found its way in perinatal care and its implementation is likely to disperse globally in the next decade [14].

Telemonitoring of fetal heart rate combined with uterine contractions in complicated pregnancies is possible with help of a wireless portable cardiotocography (CTG) system combined with a blood pressure monitor. Measurements from home are saved in a personal profile using Bluetooth. Through a secured internet portal, data are integrated in the electronic patient record system making access possible for health care professionals. In recent years, several comparable systems for remote monitoring of maternal and fetal condition have been developed and found feasible with regards to usability, acceptability and clinical usefulness [14]. As an addition to prenatal care, telemonitoring can result in increased adherence to appointments, reduced clinic visits and enhanced patient engagement [14]. However, safety of use for perinatal outcomes of these digital telemonitoring platforms has not been studied extensively in high-risk pregnancy. As an essential component in the quality of health care, patients’ involvement in the development and implementation of e-Health strategies gives relevant information to improve the use in daily practice [15].

This study aimed to assess the experiences of pregnant women during clinical hospital admission and the novelty of telemonitoring during high risk pregnancy.

## Methods

This qualitative study using online focus groups was designed as part of a pilot study for telemonitoring in high risk pregnancy. Aim of the feasibility pilot was to examine the accuracy of the tracings, the system’s usability and participants’ experiences and acceptance. In this paper we report women’s experiences of telemonitoring during the pilot.

### Context of the feasibility pilot

Wireless devices for blood pressure (Microlife WatchBP) and cardiotocography (Sense4Baby, BMA- Telenatal, The Netherlands) were used for daily follow up of patients with either PPROM, FGR or preeclampsia [16, 17]. Following a hospital admission for initial observation and treatment (e.g. antenatal corticosteroids), admitted patients were reviewed by the supervising obstetrician for eligibility for telemonitoring until start of labor. Selection criteria were 1) singleton pregnancy (for technical reasons), 2) travel time from home to the hospital within 30 min, 3) the ability to understand the devices and perform measurements as prescribed and 4) no complications requiring i.v. medication or obstetric intervention within 48 h (e.g. severe hypertension, signs of infection or antepartum hemorrhage). After instructions by a member of our centre’s Obstetric Telemonitoring Team (consisting of a clinical midwife, the resident on ward supervised by an obstetrician), participants performed their daily CTG and blood pressure before 9.30 AM. Each morning, a member of the Obstetric Telemonitoring Team reviewed the measurements and contacted her at home to ask for symptoms, discuss the results and future management. At least once a week participants visited the outpatient clinic for clinical review. In case of abnormal results (e.g. non reassuring CTG, increase in blood pressure or symptoms of hypertensive disease or infection) patients were admitted to the ward for further evaluation.

### Design

We set up online focus group (FG) discussions in secured Facebook groups within two different groups: one group of women who were admitted to the hospital during pregnancy and one group of women who were monitored at home (using home-based telemonitoring, TM).

Conducting online FG is practical to women with young children, because of the possibility to react at any time of the day while there is no time needed to travel [18]. Also, the perceived anonymity of online communication lowers social inhibitions that might hold back participants in a real-time FG. Facebook in particular is a convenient platform for an online FG, because participants are familiar with its interface, the Group function facilitates notifications, tags and commenting on comments. The Secret Group function enables privacy as participating is only possible for invitees [19].

Our FGs were conducted following a semi-structured interview protocol including open ended questions on topics that were defined after literature review and expert opinion. These included: experiences of received health care, personal feelings and family life. The groups were open to the participants and one moderator only [both research physicians, JH (male) or CT (female), trained by experienced researchers using Facebook focus groups]. The moderators did not establish a relationship with the participants before study start, expect for their occupation as researchers in the obstetric department focusing on home monitoring in pregnancy. Each focus group was open for 5 days, and two questions were posted on Facebook daily to which all women were invited to comment. When needed, the moderator commented in response to help the discussion along. All questions that were posted in the Facebook groups can be found in Additional file 1.

### Ethical approval

This study was exempted from approval of the Medical Research Ethics Committee of the University Medical Center in Utrecht (reference number 16–203), as the Committee confirmed that the Dutch Medical Research Involving Human Subjects Act (WMO) did not apply to this study.

### Sampling and recruitment

Two different groups of women were approached by phone at 6 weeks postpartum through purposive sampling. The first group consisted of women who had been admitted to our hospital for PPROM, FGR or preeclampsia, and who gave birth before the start of the telemonitoring pilot. The second group consisted of women with one of the same three complications, but who went home to receive telemonitoring during the pilot phase. Eligible candidates for the FGs had to be > 18 years, with singleton pregnancy and good ability to understand Dutch language. Those women interested in participation received written information by mail, including an informed consent form, the schedule for the study and additional information about Facebook and privacy settings. Candidates were able to ask question about the study prior to their decision to participate.

### Data collection & privacy

After receiving written informed consent, we provided additional information on how to join the discussion in a private Facebook group. All comments were saved using codes for data analysis. When the research group agreed that saturation had been reached, recruitment was stopped. Afterwards all comments were manually removed by the moderator and the Facebook groups were shut down.

### Data analysis

**Fig. 1:**
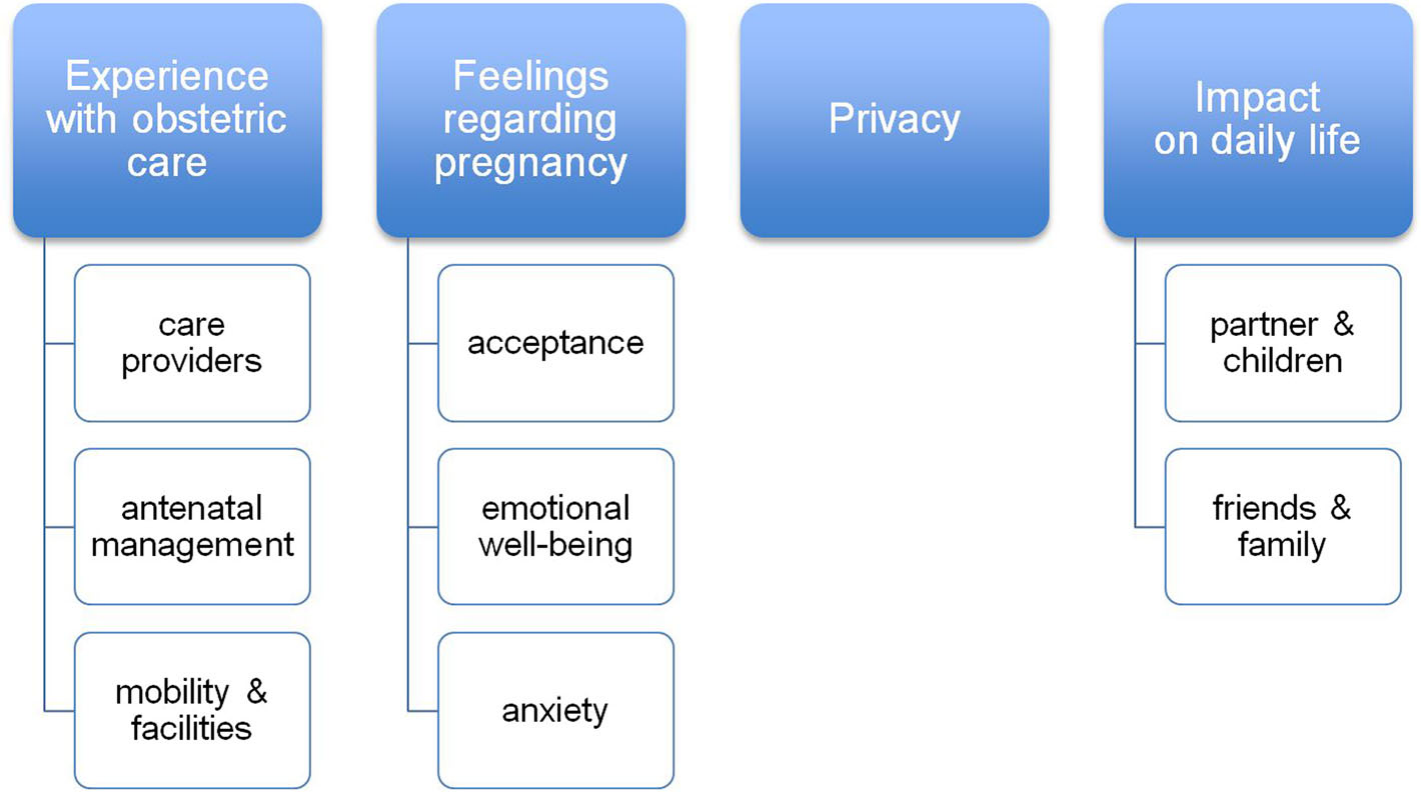
The four main themes and its subcategories resulting from the focus groups

Each step of data analysis, using an iterative and inductive process, was performed independently by JH and CT. Questions and responses were processed manually into transcripts using open coding, assigned to text fragments. After this, the initial codes were combined as they functioned as subcategories within a broader theme. Both researchers discussed the codes and grouping together ensuring accuracy of interpretation. This resulted in four themes, with three of them divided into subcategories (See Fig. 1): [1] experience with obstetric care, [2] feelings regarding pregnancy, [3] privacy and [4] impact on daily life, Participants did not provide feedback on the findings. Results were reported following the Consolidated criteria for Reporting Qualitaitve research Checklist (See Additional file 2). Representative quotes for the different themes were selected and translated into English.

## Results

Of 42 women approached, 22 women consented to participate. Reasons not to participate in the online study were: no response to the invitation, busy family life at 6 weeks postpartum, lack of Facebook account or not willing to join Facebook. We conducted four focus groups: two with participants with hospital admission in pregnancy (HA, total *n* = 11) and two with participants with telemonitoring experience in pregnancy (TM, total *n* = 11). Participant characteristics are shown in Table 1. Average length of hospital stay during admission was 17,9 days (range 7–60), average length of telemonitoring was 15,8 days (range 7–49).

**Table 1:** Participant characteristics

		Hospital admission (*n* = 11)	Telemonitoring (*n* = 11)
Age; mean (SD)		30.6 (6.6)	32.1 (5.0)
Nulliparous; n (%)		8 (72.7)	7 (63.6)
Dutch origin ^a^		9 (81.8)	8 (72.7)
Educational level ^b^	Low	2 (18.2)	2 (18.2)
	Intermediate	3 (27.2)	2 (18.2)
	High	6 (54.5)	7 (63.6)
Diagnosis; n (%)	PPROM	4 (36.4)	1 (9.1)
	FGR	3 (27.2)	7 (63.6)
	PE	4 (36.4)	3 (27.2)
Length of monitoring in days, mean (SD)		17.9 (14.9)	15.8 (12.2)
Length of monitoring in days; range		7–60	7–49

*PPROM preterm rupture of membranes, FGR fetal growth restriction, PE preeclampsia.*

^a^ Both parents born in the Netherlands (Dutch National Office of Statistics; Statistics of the Netherlands)

^b^ Education was defined as ‘low’ (elementary school, lower level of secondary school), ‘Intermediate’ (higher level of secondary school) and ‘high’ (post-secondary and university)

### Hospital admission group

#### Experience with obstetric care

Recalling their admission, half of the admitted group (6/11) was pleased with the explanation they received from the residents on ward about management and prognosis during admission. Contrarily, the others (5/11) missed coherent and straightforward management prior to their delivery, because the turnover of involved residents and obstetricians was perceived as very high during admission.

[HA10] “The general approach of management was clear to me, although it changed multiple times during admission”

[HA20] *“ … in my experience the different residents on ward constantly came up with conflicting information despite the explanation from our own ‘case manager’ [consultant obstetrician]. However, because I was admitted, it felt like contact with our case manager ceased over time, resulting in unnecessary stress and uncertainty.”*

Opinions about nurses, midwives and physicians during admission were predominantly positive. The personal approach of the nurses was highly praised, stating that questions about medical or personal issues were always possible. However, four of eleven participants addressed their concerns about the many changes of shift, causing distrust when seeing new faces every day.

[HA07] *“I was admitted during a week-end and even then we could talk to a physician and a midwife, which was very pleasant for my partner and me. The nurses were always available for a little chat.”*

[HA12] *“I had the feeling that there were many changes in residents and physicians ( … ) That’s why I felt the need to be watchful regarding my own management during admission.”*

Hospitalization often comes with restrictions in personal time and mobility, sometimes imposed by physicians, being physically bound to bed by monitors or catheters. Three participants in the hospital group remembered being in bed for hours for non-stress tests (cardiotocography), which was physically and mentally straining. The restrictions on their activity made some women very restless.

[HA11] *“I had to stay in bed or a wheelchair most of the time. Although I knew this was for the better for the baby, it was still hard on me.”*

The facilities in the hospital generated mixed reviews. Some positive remarks were made concerning the private bathrooms, television and internet, but most negative remarks were made regarding the food and beds – most of the participants missed their personal habits and choices for dinner (9/11).

#### Feelings regarding pregnancy

The majority of patients in the hospitalized group accepted the need for daily monitoring and admission, although some participants (3/11) argued that they could have stayed at home, since they did not experience any physical complaints themselves. Being confronted with pregnancy complications was mostly followed by emotions of fear, anger and sadness.

[HA07*] “We instantly understood why I had to stay in the hospital. After this, it like felt we stepped into an emotional rollercoaster … anxious, angry, sad but also relieved and happy.”*

Most of the participants in the hospital group felt bored or isolated (7/11). In some cases, boredom resulted in agitation or frustration, not knowing when or how this specific situation would end. The longer the admission lasted, the more the boredom would strike, as 4/11 subjects addressed.

[HA09] *“Later on, the boredom just intensified. It felt disturbing. Every day passed by in the same way. I could never go somewhere. Reading and watching television is only amusing for a while, but not the whole day, each and every day.”*

The presence of anxiety and fear is associated with the uncertainty of the future health of their babies or their own body. Admitted participants felt more anxious as the admission continued, since they heard more about the risks associated with high risk pregnancy. Worrisome results of ultrasounds, cardiotocography or blood tests altered these feelings of fear. On the contrary, experiencing calm periods in the hospital, or hearing promising results of antenatal tests was beneficial. One participant [HA11] raised the concept that her stay in the hospital felt safe, “… knowing that personnel was close by and able to react quickly in acute situations.”

#### Privacy

In the hospitalized group, the subject of privacy generated strong reactions during our study. As the ward is a relatively public area, staff, other patients, their family, friends can move in and out of the room at almost every time of the day. This interfered with personal routines and privacy.

[HA19] *“I really missed my privacy. Anyone in my room could overhear the chats I had with family and friends, which was really annoying.”*

[HA21] *“There was a lack of privacy. While overhearing everything my ‘roommate’ said, she could also hear my talking. I didn’t feel comfortable while talking to the doctors while she was in the room. Visits of my partner didn’t really feel like we were there together.”*

[HA09] *“How would you feel if everything you discuss with your doctor, can be heard by all your roommates on ward? I didn’t want to share all this personal information with strangers.”*

A discussion arose on the positive and negative sides of rooms shared with multiple patients. Half of the hospitalized group felt they found support in contact with their roommate. Others were bothered with their neighbors and their visitors, only divided by a curtain.

[HA19] *“A curtain doesn’t mean there is any privacy. I missed having personal conversations with family. But the last two days I was in a private room and I missed the amusement and relaxation of being with other patients. A compromise would be great.”*

#### Impact on daily life

The need for support from loved ones was mentioned several times by the admitted patients. Although family members and friends were able to visit the ward, the impact on them is not to be underestimated. Partners often worked normal working hours during admission and spent much time traveling to and from the hospital, which felt difficult and tiresome. Pregnant women missed spending time with their other children and partners (8/11).

[HA08] *“It had a big impact on my two-year-old son. We had never been separated before and saying goodbye to him was hard, every single day. It was also hard on my partner. He suddenly had to take care of our child on his own, take time off from work and drive to the hospital, sometimes 2-3 times a day.”*

### Telemonitoring

#### Experience with obstetric care

The home-based telemonitoring started with an elaborate explanation of the care-pathway and the use of the monitoring equipment. The devices were easy to use and participants had no to very little technical issues (10/11). The daily results and plans for future antenatal management were discussed by phone by the Obstetric Telemonitoring Team members.

[TM03] *“ … [the midwife] explained the use of the equipment and took us through all the steps of the entire process. For me it was really nice to speak to somebody on the phone every single day. In my experience they would call quickly after sending the CTG and they would take the time to answer all of my questions.”*

The weekly appointments in the outpatient clinic during TM were often appreciated (7/11), although, some women experienced getting back and forth from the hospital as a hassle. All highly valued the daily contact with the Obstetric Telemonitoring team, primarily midwives. By telephoning each day, they had the chance to ask their questions and be reassured if needed. The clinical midwives of the Obstetric Telemonitoring Team were often described as ‘empathetic’ and ‘very competent’.

[TM01] *“I really appreciated the daily phone calls, and when things got to my head, the midwife functioned as a sympathizing listener ( … ) They were really helpful.”*

In general, mobility did not seem to be a pressing issue for the women at home, even though some of the women were told to rest as much as possible. Being home-based, all the women agreed that sleeping in their own bed was much more comfortable than a hospital bed, waking up well- rested. Those women, who were admitted first and subsequently received telemonitoring, added that the noises on ward during night had profound impact on their sleep, which was not the case at home.

#### Feelings regarding pregnancy

In general, all of the telemonitoring women agreed that the indication for daily monitoring was clear. Again, the complications caused feelings of uncertainty, anxiety and restlessness.

[TM05] *“We understood the reasons for all the extra assessments during pregnancy. It gave us a safe and reassuring feeling, knowing that somebody kept an eye on the baby on a daily basis, and that our concerns were taken seriously. “*

[TM03] *“I am very happy that I didn’t need to stay in the hospital for weeks, but that I was offered telemonitoring instead.”*

The women in telemonitoring enjoyed being at home. They expressed being at home was more peaceful and calm then being admitted at the hospital (10/11). Even though these women were not admitted in a hospital, they were very well aware that their pregnancy was complicated.

[TM03] Because I had to stop working during the 30th week of my pregnancy, as I had to monitor myself every morning, I felt like a high-risk patient.

Only 2 out of 11 women from this group expressed anxious episodes at home. In these two subjects, anxiety was related to the realization of being alone when trying to do the correct monitoring or the need to come to the hospital for further evaluation. This uncertainty sometimes caused concern about what was going to happen next.

[TM07] *“Yes, sometimes I would have preferred to have a doctor nearby, for example if I could not find the fetal heart rate with the monitor or when I did not feel the baby move for quite a while.”*

[TM16] *“I enjoyed being at home; it was a lot better than being in the hospital ( … ) I had to return to the hospital multiple times because of my blood pressure or a questionable CTG. Each time this happened, I had to wait and see if I could go back home. This made me feel like a high risk patient, although much less then when I was admitted to the hospital.”*

#### Privacy

In contrast to the hospital based participants, none of the women in the telemonitoring group reported issues regarding privacy.

#### Impact on partner and/or family

Being at home resulted in less travel time for partners or family for hospital visits, which had its positive effects on family life. Some women in the telemonitoring group had help at home (3/11): family would help out with errands, house cleaning or taking care of other children. One woman [TM2] pointed out that, having a toddler around, resting at home was not always easy.

[TM1] Daily life just continued when I was at home, for both me and my family… In the morning, I sent the CTG, and I rested a little more than I would normally do, because of my blood pressure.

[TM2] Home monitoring was much more relaxed to me, compared to my first pregnancy when I was admitted in my 29th week. Now I could stay with my little son, very important for both his comfort and mine.

## Discussion

The aim of this qualitative study was to compare the experiences of high risk pregnant women during hospital admission or telemonitoring. Although the uncertainty of high risk pregnancy remains an intense experience for both women and their families, telemonitoring seems to allow them to experience this considerably less stressful compared to hospital admission.

Complications of pregnancy come with feelings such as fear and frustration, especially while being admitted to the hospital. As antenatal anxiety and depressive symptoms are common among obstetric inpatients, they increase the risk of post partum depression and adversely affect infant and child development [20]. The hospital admission group in our study reported a growing sense of boredom and anxiety during their admission, which is in line with earlier work on hospitalization during pregnancy: women report concerns for the health of their future baby, feeling of helplessness and loneliness while being separated from home, family and friends [10–12]. Lack of privacy, when admitted, affected our patients’ contact with health care providers, partner, kids (if present) and other family and friends. In contrast, the experiences at home in our telemonitoring group were more positive: although they still felt like a patient at times, the TM group responded that the comfort of their own home and bed was very pleasant. In this group, only a minority of participants reported being anxious at times at home, while not having a physician or nurse nearby. Findings from our and previous studies reveal that telemedicine could provide important psychological benefits during pregnancy [21]. When women’s perception of high risk pregnancy and quality of care experience improve with telemonitoring, this may contribute to an increase in quality of life and reduction of antenatal anxiety and its consequences for mother and child.

O’Brien et al. and Rauf et al. described the experiences of remote fetal monitoring during outpatient induction of labour in a low risk pregnancy group in 2009–2010 [22, 23]. Their study made use of wireless fetal-maternal monitoring device for remote non-invasive trans-abdominal monitoring of fetal heart activity, and electromyography for uterine activity. The participants concluded that telemonitoring during induction offered them freedom and familiarity of home environment, but feelings of reassurance depended on effective communication with hospital staff. These observations are in line with our findings, as our participants reported a positive effect of staying at home while being monitored daily by familiar midwives within the Obstetric Telemonitoring Team, opposed to tridaily changes in hospital staff on maternity ward. Also in remote blood pressure monitoring during pregnancy, women report the willingness to participate in self-monitoring strategies [24]. Women experiences feeling of safety, because their home measurements were monitored in clinic by health care providers, taking action when needed.

The experiences of our telemonitoring group correspond with trends of eHealth use in perinatal care: women of reproductive age are interested in e-Health, because of their frequent use of smartphone, apps, and online searches for pregnancy education [14]. Literature reviews conclude that health outcomes for eHealth interventions in perinatal care are generally positive, resulting in lifestyle and mental health improvement or providing multiple other advantages while health outcomes were found equal (e.g. in gestational diabetes) [14, 25, 26].

Social changes are demanding a shift to home-based patient-centered care, and remote monitoring provides flexibility to both physicians and patients to decrease the demand for more hospital personnel or clinic space [27]. Both groups embrace telemedicine because of its usability, tendency to improve access to care, communication and outcomes while decreasing clinic visits and travel time [28]. These changes are assumed to have profound cost-saving effects in favor of telemonitoring, an important aspect regarding the ever-increasing health care costs – and workloads [29]. Compared to usual care, possible additional time associated with telemonitoring (instructions for patients, daily telephone contact, and weekly outpatient visits) should be explored in cost-effectiveness studies. Organizations will potentially benefit from telehealth as it decreases missed appointments, waiting times and re-admissions, although reimbursement lacks to progress due to legislation and swift technological advancements.

Implementation of (fetal) telemonitoring in pregnancy is not studied extensively, and further research is needed on the effectiveness on both health outcomes and costs of this innovative strategy. Furthermore, not much is known about the ethical considerations that are necessary for successful implementation [30]. Incorporating patients’ preference is important to ensure that care is provided based on the individual patient’s perspective, preferences, and needs. The findings of this study provide some suggestions for implementation from the patient perspective: these include the demand for patient education and a clear antenatal management plan, adequate participant selection for telemonitoring, daily contact (by telephone or teleconferencing) by a select group of staff for a continuum of care (as our Obstetric Telemonitoring Team) and weekly hospital visits. Regarding safety, it is recommended to work using strict protocols including equipment manuals for care providers and patients and a limit for travel time to the hospital.

This study adds to the current knowledge on women’s perspectives on antenatal monitoring from home during high-risk pregnancy. A strength of this qualitative study is the inclusion of both hospitalized women and women from the telemonitoring pilot within one center. Although there is existing knowledge of personal effects of hospitalization during pregnancy, these effects can differ due to different protocols of daily practice in different hospitals, for example visitation policies on ward, the number of private and shared rooms and other hospital facilities. By directly comparing both groups from our center, we were able to outline the different experiences and perspectives in these two groups.

Our results must be interpreted in the context of the following limitations. Selection bias could have influenced the results, as participants of telemonitoring agreed to take part in this innovative strategy. Although findings from the focus groups were seemingly consistent, the results are not statistically powered. This study uses qualitative methods and thus provides mainly descriptive data that cannot be generalized widely. Although there are benefits of online FG’s as described, the asynchronous nature of this FG method could have had its effect on the discussions between participants.

## Conclusions

Telemonitoring of a high-risk pregnancy provides an innovative manner to monitor fetal and maternal condition from home. Compared to the experiences of hospital admission in high risk pregnancy, it allows women to be in a private and comforting environment during an anxious time in their lives. As future studies should further investigate the safety and cost effectiveness of this innovative strategy, women’s views on the preference of telemonitoring need to be taken into consideration.

## Supplementary information

**Additional file 1.** Overview of questions posted in the online Facebook groups as part of the qualitative study.

**Additional file 2.** COREQ (COnsolidated criteria for REporting Qualitative research) Checklist.

## Data Availability

The dataset used and analysed during the current study are available from the corresponding author on reasonable request.

## Abbreviations

FG: Focus group
FGR: Fetal growth restriction
HA: Hospital admission
PROM: Preterm rupture of membranes
TM: Telemonitoring
