# Supplementary material for: Home-based telemonitoring versus hospital admission in high risk pregnancies: a qualitative study on women’s experiences
Source: BMC Pregnancy Childbirth. 2020 Feb 4;20:77. doi: 10.1186/s12884-020-2779-4 (PMC7001237; doi:10.1186/s12884-020-2779-4)
Supplement: Supplementary file 1 — Additional file 1. Overview of questions posted in the online Facebook groups as part of the qualitative study. [file 12884_2020_2779_MOESM1_ESM.docx]

**Focus group questions “Hospital admission”**

Question 1:

Was it clear to you (and, if applicable, your partner) why hospital admission was necessary during your pregnancy? How did you feel about the admission, at first?

Question 2:

During your admission, was the management plan regarding your complicated pregnancy clear to you? And was it possible to speak to as physician, midwife or nurse in an accessible manner?

Question 3:

Can you tell us about your thoughts about the admission; did you feel it was necessary or useful?

Question 4:

Can you tell us about the effects of our hospitalization on your partner or family at home, if applicable? How did they manage with work, school, family and your admission?

Question 5:

The longer your admission lasted, did you recognize a change in perception? For exemple, did you feel more relaxed, or bored, or more anxious?

Question 6:

During admission, how did you feel about your freedom of movement and your daily activities? Was it possible to move around, or were you confined to bed, or bed rest?

Question 7:

Can you tell us more about privacy? Were you able to find a place for you alone on ward? Did you manage to do things for yourself?

Question 8:

Can you tell something about hospital facilities, such as food, television, beds?

Question 9:

Is there something you feel you want to share with us, in ways of points of attention or negative points about hospital admission?

Question 10:

Is there something you feel you want to share with us, in ways of the positive points about hospital admission?

**Focus group questions “Telemonitoring”**

Question 1:

Was it clear to you (and, if applicable, your partner) why increased monitoring was necessary during your pregnancy? How did you feel about this increased surveillance, at first?

Question 2:

During your period of telemonitoring, was the management plan regarding your complicated pregnancy clear to you? And was it possible to speak to a physician, midwife or nurse in an accessible manner?

Question 3:

Can you tell us about your thoughts about the weekly outpatient visits during telemonitoring; did you feel it was necessary or useful?

Question 4:

How are your experiences with use of the devices at home? Was it easy to use, or did the devices or technique let you down sometimes?

Question 5:

How did you combine home-based telemonitoring with life at home? Did you feel like you were still ‘admitted’? Or did your daily life (if applicable with your partner and other kids) just continue as normal?

Question 6:

The longer your telemonitoring period lasted, did you recognize a change in perception? For example, did you feel more relaxed, or bored, or more anxious?

Question 7:

When your experienced times of insecurity or anxiety, did you feel it would have helped if a nurse/midwife/physician would have been around, as would be the case on the hospital ward?

Question 8:

Can you tell something about facilities at home, compared to the facilities in the hospital or ward (if you have ever been admitted)?

Question 9:

Is there something you feel you want to share with us, in ways of points of attention or negative points about home-based telemonitoring?

Question 10:

Is there something you feel you want to share with us, in ways of the positive points about home-based telemonitoring?
